# Genome-Wide Identification and Expression Analysis of the Cys2His2 Zinc Finger Protein Gene Family in *Flammulina filiformis*

**DOI:** 10.3390/jof10090644

**Published:** 2024-09-11

**Authors:** Zongjun Tong, Xing Han, Xinlian Duan, Junbin Lin, Jie Chen, Jihong Xiao, Ying Gan, Bingcheng Gan, Junjie Yan

**Affiliations:** 1 Institute of Urban Agriculture, Chinese Academy of Agricultural Sciences, Chengdu 610000, China; ttzjun1@163.com (Z.T.); hanxing@caas.cn (X.H.); duanxinlian@caas.cn (X.D.); linjunbin@caas.cn (J.L.); chen-jiespring@outlook.com (J.C.); xiaojihong05@163.com (J.X.); ganying@caas.cn (Y.G.); 2 Chengdu Agricultural Science and Technology Center, Chengdu 610095, China

**Keywords:** C2H2 zinc finger proteins, *Flammulina filiformis*, gene expression, phylogenetic analysis, fungal development

## Abstract

Zinc finger proteins (ZFPs) are essential transcription factors in eukaryotes, particularly the extensively studied C2H2 family, which is known for its involvement in various biological processes. This research provides a thorough examination and analysis of the C2H2-ZFP gene family in *Flammulina filiformis*. Using bioinformatics tools, 58 *FfC2H2-ZFP* genes spread across 11 chromosomes were identified and scrutinized in detail for their gene structures, protein characteristics, and phylogenetic relationships. The study of phylogenetics and synteny sheds light on the evolutionary relationships among C2H2-ZFPs in *F. filiformis* and other fungi, revealing a complex evolutionary past. The identification of conserved cis-regulatory elements in the gene promoter regions suggests intricate functionalities, particularly in the developmental and stress response pathways. By utilizing RNA-seq and qRT-PCR techniques, the expression patterns of these genes were explored across different developmental stages and tissues of *F. filiformis*, unveiling distinct expression profiles. Notably, significant expression variations were observed in the stipe elongation region and pilei of various sizes, indicating potential roles in fruiting body morphogenesis. This study enhances our knowledge of the C2H2-ZFP gene family in *F. filiformis* and lays the groundwork for future investigations into their regulatory mechanisms and applications in fungal biology and biotechnology.

## 1. Introduction

Zinc finger proteins (ZFPs) are a prominent family of transcription factors found in eukaryotes [1]. They are characterized by their distinct zinc finger functional domains, which consist of cysteines (Cys) and/or histidines (His) [2]. The ZFP family is further categorized into various groups based on the presence of Cys and His, such as C2H2, C8, C3HC8, and C2HC, among others. Miller et al. first discovered C2H2-ZFPs in 1985 [3], and their research remains one of the most extensively studied transcription factors to date. The typical structure of this motif is X2-C-X2,4-C-X12-H-X3,4,5-H (where X represents any amino acid, C denotes Cys, H indicates His, and the numbers denote the quantity of amino acids) [4]. These transcription factors are essential for mechanisms related to growth, development, and stress responses in animals [5], plants [6], and fungi [7].

At present, C2H2-ZFPs have been extensively studied in various plants, including rice [8], wheat [9], apple [10], sorghum [11], cotton [12], and strawberry [13]. These studies have confirmed the significant role of C2H2-ZFPs in plant growth, development, and resistance to abiotic stress [14,15,16,17]. Similarly, C2H2-ZFPs have notable effects on fungal mycelia growth, spore development, fruiting body formation, and stress response. For instance, the *vadH* gene has been shown to regulate both asexual and sexual development in *Aspergillus nidulans* [18]. In *Metarhizium acridum*, the Δ*MaNCP1* strain can notably decrease the conidial yield, delay conidia germination and mycelial growth, and reduce the tolerance to UV-B irradiation and heat stress [7,19]. Additionally, some C2H2-ZFPs have been reported in edible fungi. The *PuCRZ* gene has been found to positively regulate the response of *Polyporus umbellatus* mycelium to osmotic stress [20], while the overexpression of the gene *c2h2* can result in a one-day advancement in the peak daily yield of *Agaricus bisporus* [21]. Furthermore, the dual mutation strain Δ*c2h2*Δ*c2h2* of *Schizophyllum commune* is unable to form fruiting bodies [22]. In *Coprinopsis cinerea*, the absence of the exp2 gene leads to a phenotype with no cap expansion [23]. To date, the complete identification of the C2H2-ZFP gene family within the genomes of edible fungi has been limited to the species *Pleurotus ostreatus* [24] and *Ophiocordyceps sinensis* [25].

*Flammulina filiformis* is a widely cultivated and well-known edible fungus, valued for its nutritional and medicinal benefits [26]. Its short and consistent cultivation cycle makes it an ideal model organism for studying the growth and development of fruiting bodies in edible fungi [27]. Some molecular mechanisms governing the development of the fruiting body in *F. filiformis* have been researched. The overexpression of the *Ste12-like* gene can increase the number of fruiting bodies [28], while the expression of the *FfJMHy* gene is linked to stipe elongation [29]. The elongation of the stipe in *F. filiformis* is controlled by reactive oxygen species (ROS) signaling molecules, with high carbon dioxide levels inhibiting pileus development [27]. Previous studies have suggested that members of the C2H2-ZFP gene family exhibit differential expression in the different stipe regions of *F. filiformis* [30], although the entire C2H2-ZFP gene family has not been identified in the genome of this species. This paper aims to provide a comprehensive analysis of the C2H2-ZFP gene family in *F. filiformis*.

In this study, we identified 58 C2H2-ZFP genes and conducted a comprehensive analysis of gene structure, protein characteristics, conserved structural domains, cis-acting elements, and phylogenetic relationships. Furthermore, we investigated the expression patterns of these genes in various parts of the fruiting bodies of *F. filiformis* according to the RNA-seq data. Additionally, we examined their expression levels in the different regions of the stipe and pilei of various sizes using qRT-PCR. These findings enhance our understanding of the C2H2-ZFP transcription factor gene family in *F. filiformis* and offer a reference for future research on the role of C2H2-ZFPs in the growth and development of *F. filiformis*, thereby providing potential target genes for the breeding of superior varieties.

## 2. Materials and Methods

### 2.1. Identification and Chromosomal Localization of C2H2-ZFPs in F. filiformis

The *F. filiformis* genome sequence was obtained from the BioProject: PRJNA603211 at the National Center for Biotechnology Information (NCBI). To identify potential C2H2 zinc finger proteins in the *F. filiformis* genome, a Hidden Markov Model (HMM) profile of the C2H2 zinc finger protein domain (PF00096) from the Pfam database (https://www.ebi.ac.uk/interpro/entry/pfam/, accessed on 27 February 2024) was used to search the entire proteome sequence with HMM searcher 3.0. Sequences with an E-value of less than 0.01 were considered as potential C2H2-ZFPs in *F. filiformis*. Subsequently, these sequences were analyzed using the SMART database (https://smart.embl.de/, accessed on 29 February 2024) to confirm the presence of the C2H2 zinc finger domain (SM00355). The predicted FfC2H2-ZFPs were further analyzed for protein features using the EMBOSS Pepstats tool of EMBL-EBI (https://www.ebi.ac.uk/jdispatcher/seqstats/emboss_pepstats, accessed on 29 February 2024) and for subcellular localization using WoLF PSORT (https://wolfpsort.hgc.jp/, accessed on 29 February 2024). Finally, the chromosomal locations of the *C2H2-ZFP* genes were mapped onto an *F. filiformis* genome map using TBtools-II [31].

### 2.2. Gene Structure and Conserved Motif Analysis

To analyze the exon and intron structures of the *FfC2H2-ZFP* genes, the gene information was entered into the ‘Gene Location Visualize (Advanced)’ tool in TBtools-II [31]. Additionally, MEME 5.5.5 (https://meme-suite.org/meme/tools/meme, accessed on 8 March 2024) was utilized to identify the motifs within the FfC2H2-ZFPs [32]. The analysis was conducted with the following parameters: motif sites distribution with any number of repetitions, the identification of 5 motifs, and the minimum and maximum motif widths of 6 and 50.

### 2.3. Phylogenetic Analysis of C2H2-ZFPs in F. filiformis

The protein sequences of the C2H2-ZFPs from *F. filiformis* (GenBank accession numbers: PQ279402 to PQ279459), *A. bisporus* (downloaded from the Fungal Transcription Factor Database at http://ftfd.snu.ac.kr/, accessed on 16 May 2024 [33]), *Laccaria bicolor* (also downloaded from the Fungal Transcription Factor Database), *P. ostreatus* [24], and *Armillaria ostoyae* (PRJNA635555) were analyzed phylogenetically. Sequence alignment was conducted using the MUSCLE algorithm, followed by trimming with trimAl. A phylogenetic tree was constructed using the IQTREE application with the maximum likelihood method and supported by a bootstrap value of 1000. The tree was then visualized and annotated on the iTOL platform (https://itol.embl.de/, accessed on 5 July 2024) for better clarity. Furthermore, a separate phylogenetic tree was created, specifically using the C2H2-ZFP protein sequences from *F. filiformis* to enable more detailed analysis.

### 2.4. Cis-Element Analysis of C2H2-ZFPs in F. filiformis

The 2000 bp sequences located upstream of the translation start site (TSS) for the *FfC2H2-ZFP* genes were obtained from the genome of *F. filiformis* using TBtools-II [31]. The PlantCARE database (https://bioinformatics.psb.ugent.be/webtools/plantcare/html/, accessed on 15 March 2024) was utilized to identify the cis-regulatory elements present in the promoters of *FfC2H2-ZFP* genes. Following this, the findings were visually represented using the Gene Structure Display Server 2.0 tool (https://gsds.gao-lab.org/, accessed on 28 June 2024) [34].

### 2.5. Gene Duplication and Ka/Ks Analysis

Gene collinearity analysis was conducted using the default parameters of MCScanX [35] to explore the collinearity among *F. filiformis* and other edible fungi species, including *A. bisporus* (PRJNA635555), *Lentinula edodes* (PRJNA627073), *P. ostreatus* (PRJNA933115), and *A. ostoyae* (PRJEB19205), as well as within *F. filiformis* itself. The results were visually presented using TBtools-II [31], which also enabled the calculation of Ka/Ks ratios for the identified collinear genes.

### 2.6. Expression Pattern Analysis

To analyze the expression patterns of the *FfC2H2-ZFP* genes in various tissues, we obtained transcriptome raw data from the NCBI for six tissues, the vegetative mycelium (VM), the primordium (PR), the young fruiting body cap (YFBC), the fruiting body cap (FBC), the young fruiting body stipe (YFBS), and the fruiting body stipe (FBS), under the project number PRJNA557510 [36]. We processed the data by filtering out low-quality information using fastqc, aligning the sequencing data to the *F. filiformis* genome with HISAT2 [37], calculating the count values with HTSeq-count [38], and normalizing the count values to FPKM using Perl script, all with the default parameters. Subsequently, we extracted the FPKM values for the *FfC2H2-ZFP* genes and generated a heatmap using TBtools-II [31].

### 2.7. Strains and Collection of Samples

A white-fruit dikaryotic strain of *F. filiformis*, designated as Fv01, was obtained from the Fujian Edible Fungi Germplasm Resource Collection Center in China. The culture media for both the mycelium and fruiting bodies were adopted from the methodology outlined by Yan et al. [39], and the cultivation techniques for fruiting bodies were based on the findings of Park et al. [40]. Samples were collected from the stipe elongation region (ER) and the stable region (SR) following the protocols described by Yan et al. [30]. Additionally, samples of the pilei at different developmental stages (3 mm, 9 mm, 15 mm, 21 mm, and 33 mm) were obtained during the growth of *F. filiformis*. These specific sizes correspond to distinct morphological and physiological changes, reflecting the maturation process of the fruiting body. For example, smaller samples at the initial stages of growth demonstrate rapid cellular division and tissue expansion, while larger samples at the later stages indicate structural stabilization and the transition toward metabolic activities that support spore production and dispersal. Each sample was collected in triplicate and preserved in liquid nitrogen.

### 2.8. Gene Expression Analysis

Total RNA was extracted from the collected samples using the MagPure Plant RNA Kit B (Magen Biotechnology, Guangzhou, China). Subsequently, first-strand complementary DNA (cDNA) was synthesized from the isolated total RNA using the TransScript All-in-One First-Strand cDNA Synthesis SuperMix for qPCR (TransGen Biotech, Beijing, China). Quantitative real-time PCR (qRT-PCR) was conducted with the QuantStudio 1 Real-Time PCR System (Thermo Fisher Scientific, Waltham, MA, USA), utilizing the TransStart Top Green qPCR SuperMix (TransGen Biotech, Beijing, China) for fluorescence detection. For normalization, two endogenous control genes, glyceraldehyde-3-phosphate dehydrogenase (*FfGAPDH*) and Ras-related small GTPase (*FfRas*), were selected to serve as reference genes [41]. The primer sequences (Table S1) for the target genes were meticulously designed employing the Primer-BLAST tool (https://www.ncbi.nlm.nih.gov/tools/primer-blast/, accessed on 8 July 2024). The relative expression levels of the target genes were quantified using the 2^−∆∆Ct^ method [42]. Statistical analysis was performed using GraphPad Prism 9.0 software (GraphPad Software, San Diego, CA, USA).

## 3. Results

### 3.1. Identification of C2H2-ZFP Gene Family in F. filiformis

A total of 60 C2H2-ZFPs were identified in *F. filiformis* using the HMM searcher 3.0 with an E-value threshold of less than 0.01. Following this, the retrieved sequences were searched against the SMART database, and each C2H2 zinc finger domain was manually examined. Only the protein sequences containing the conserved motif ‘C-X2,4C-X12-H-X3,4,5H’ were retained. In summary, 58 C2H2-ZFPs, each containing structural domains characteristic of the C2H2 zinc finger, were found in the *F. filiformis* genome. These genes ranged from 144 to 961 amino acids in length, with an average of 389 amino acids. The protein sequences’ molecular weights were predicted using the EMBOSS Pepstats tool, with values ranging from 15,976.16 to 103,900.77 Daltons (Da) and an average molecular weight of 43,115.33 Da. Subcellular localization analysis with WoLF PSORT revealed that all these proteins are localized to the nucleus, which aligns with their role in binding to the DNA promoter regions (Tables S2 and S3).

### 3.2. Chromosomal Distribution and Gene Duplication in the FfC2H2-ZFP Genes

**Figure 1:**
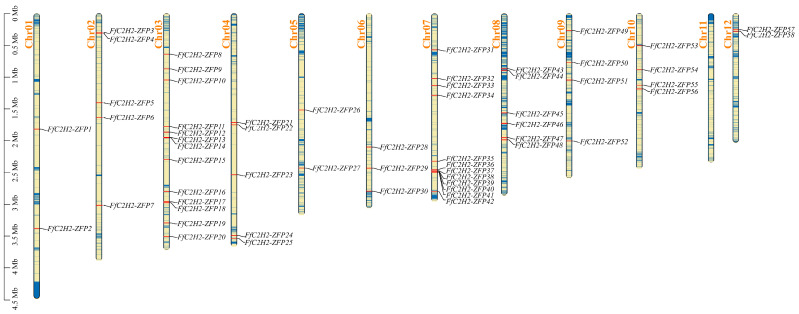
Chromosomal distribution of the *FfC2H2-ZFP* genes in *F. filiformis* genome. Chromosomal length is given in Megabases (Mb), and the *FfC2H2-ZFP* genes are highlighted in black.

To investigate the chromosomal distribution of the *FfC2H2-ZFP* genes, we utilized the whole genome sequence of *F. filiformis* for localization analysis, employing TBtools-II [31] to effectively visualize and present the data. Our findings revealed clear chromosomal locations for all the *FfC2H2-ZFP* genes, with a total of 58 genes dispersed across 11 chromosomes, except for chromosome 11. The distribution of the *FfC2H2-ZFP* genes showed non-uniformity across the different chromosomes and exhibited no correlation with the chromosome length. Notably, chromosome 1, the longest chromosome, only contained two genes, while chromosome 3, a relatively shorter chromosome, housed thirteen genes. Distribution patterns were not apparent, although gene clusters were observed on chromosomes 3 (two genes) and 7 (five genes) (Figure 1). Furthermore, synteny analysis revealed the absence of collinear pairs of the *FfC2H2-ZFP* genes within the genome (Figure S1).

### 3.3. Phylogeny Analysis, Motifs Assay, and Gene Structure of the FfC2H2-ZFPs

**Figure 2:**
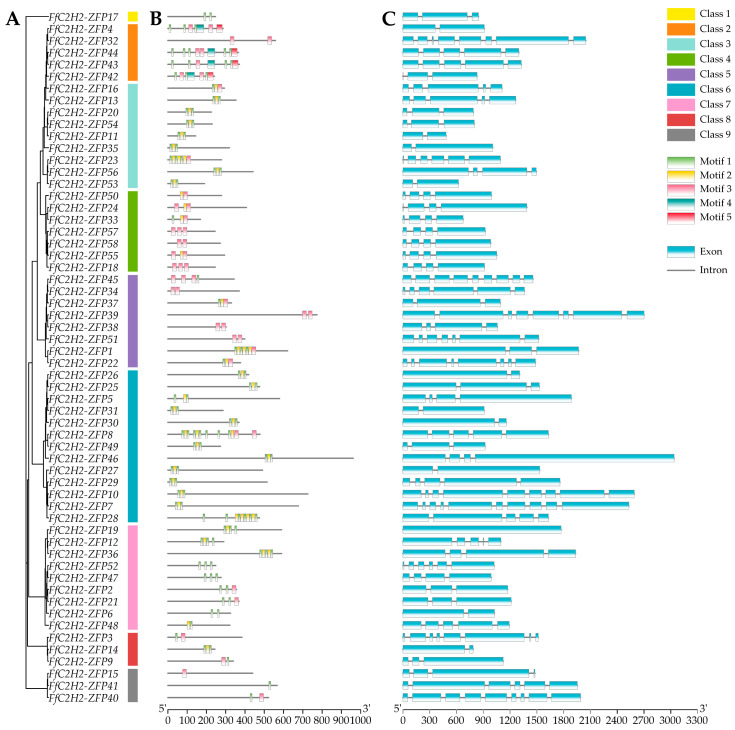
The analysis of the FfC2H2-ZFP genes in *F. filiformis*. (**A**) A phylogenetic tree was constructed using the maximum likelihood (ML) method, with a bootstrap value of 1000. (**B**) The conserved motifs (1–5) in the FfC2H2-ZFPs are depicted in different colors, with the gray lines indicating the relative protein length. (**C**) The gene structure is illustrated with exons as green rectangles and introns as gray lines.

Phylogenetic analysis was performed to elucidate the evolutionary relationships among the FfC2H2-ZFPs. A maximum likelihood (ML) tree was constructed based on their protein sequences, with a bootstrap value of 1000. The FfC2H2-ZFPs were categorized into nine classes (1–9) with varying gene numbers: class 1 (one gene), class 2 (five genes), class 3 (nine genes), class 4 (seven genes), class 5 (eight genes), class 6 (thirteen genes), class 7 (nine genes), class 8 (three genes), and class 9 (three genes) (Figure 2A). The protein sequences of all 58 FfC2H2-ZFPs were subjected to motif analysis using MEME, revealing five conserved motifs (Figure S2). Motifs 1, 2, and 3 corresponded to the C2H2 conserved structural domains. Notably, all 58 FfC2H2-ZFPs contained at least one of these motifs, with motif 1 present in 46 genes (Figure 2B). This indicates the potential importance of motif 1 as a conserved structural domain in *F. filiformis*. Additionally, motifs 4 and 5 were exclusively identified in four genes within group 2. The similarity in motif composition aided in grouping the genes together, supporting phylogenetic analysis. Further analysis focused on the structural features of the *FfC2H2-ZFP* genes, particularly comparing the intron and exon compositions. Most genes exhibited varying numbers of exons (ranging from one to ten) (Figure 2C). The exon–intron patterns aligned with the phylogenetic groupings, suggesting a potential link between the exon–intron structures and the biological functions of the FfC2H2-ZFPs.

**Figure 3:**
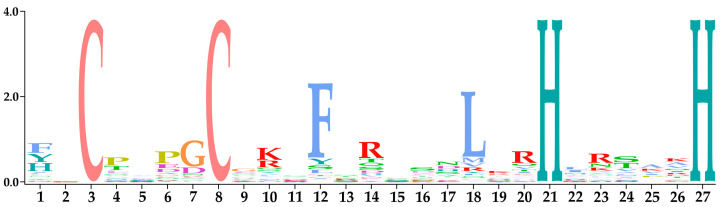
The conserved amino acid analysis of the C2H2 domain in *F. filiformis*. The height of each amino acid represents its conservation ratio.

To analyze the structural domain characteristics of the C2H2-ZFPs in *F. filiformis*, the conserved structures were compared using multiple sequence alignments facilitated by Jalview software 2.11 [43]. Analysis revealed that the 58 FfC2H2-ZFPs contained 129 C2H2 domains, each consisting of 23–26 amino acids (Figure S3). Each domain was characterized by two cysteines and two histidines, with phenylalanine and leucine also showing relative conservation. The conserved structural domain (CX2-4CX12HX3-5H) was collectively formed by these residues, showcasing a significant degree of conservation within the FfC2H2-ZFPs (Figure 3).

### 3.4. Cis-Element Analysis of the C2H2-ZFP Gene Family in F. filiformis

**Figure 4:**
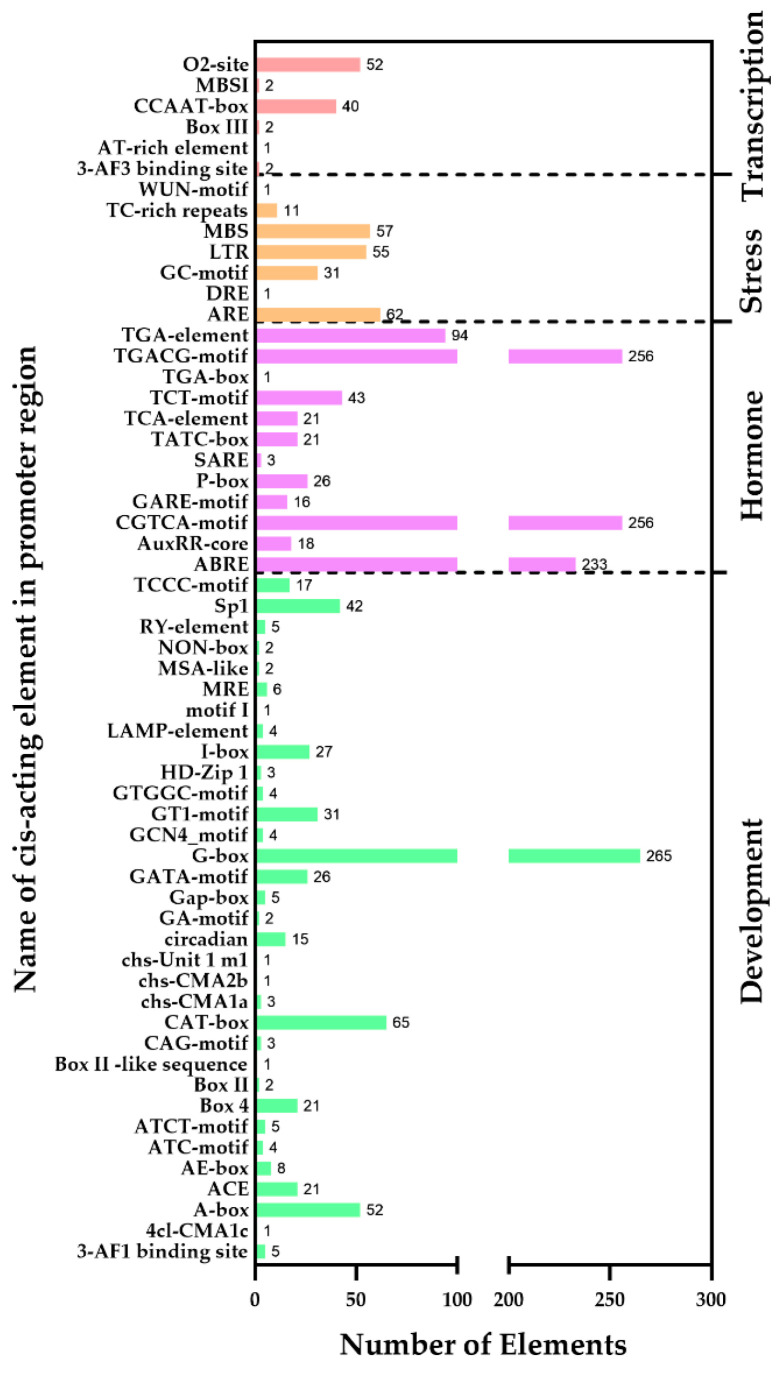
Cis-element analysis of the promoters of the *C2H2-ZFP* genes in *F. filiformis*.

Promoters play a crucial role in gene expression regulation. To investigate the function of the FfC2H2-ZFPs, the 2000 base pair (bp) upstream sequence of the *FfC2H2-ZFP* genes was analyzed as a putative promoter, with its cis-elements examined using PlantCARE. The distribution and function of these elements were subsequently studied (Figure S4). Comprehensive analysis revealed a total of 1954 cis-acting elements distributed across 58 different types within the *FfC2H2-ZFP* genes (Figure 4). Among these, the elements related to development were the most prevalent, with 654 instances across 33 types, constituting 33.38% of the total identified cis-acting elements. Notable among these are the G-, CAT-, and A-box elements, underscoring the significance of the FfC2H2-ZFPs in the growth and development of *F. filiformis*. Additionally, the hormone-related elements were prominent, with 988 instances across 12 types, accounting for 50.43% of the total instances. These include motifs such as CGTCA, TGACG, ABRE, and TGA elements, indicating a likely role for the FfC2H2-ZFPs in modulating plant hormones, including abscisic acid (ABA), methyl jasmonate (MeJA), and auxin. Furthermore, 218 instances of seven types of cis-acting element were associated with stress responses, comprising 11.13% of the total instances. These elements, such as ARE, MBS, and LTR, suggest that the C2H2-ZFPs are implicated in the responses of *F. filiformis* to various stresses, including anaerobic conditions, drought, and low temperatures. The remaining 99 instances of six types of cis-acting elements, representing 5.05% of the total instances, are primarily linked to transcriptional regulation, further highlighting the multifaceted regulatory roles of the FfC2H2-ZFPs within the genomic landscape of *F. filiformis*.

### 3.5. Phylogenetic Analysis of the FfC2H2-ZFPs

**Figure 5:**
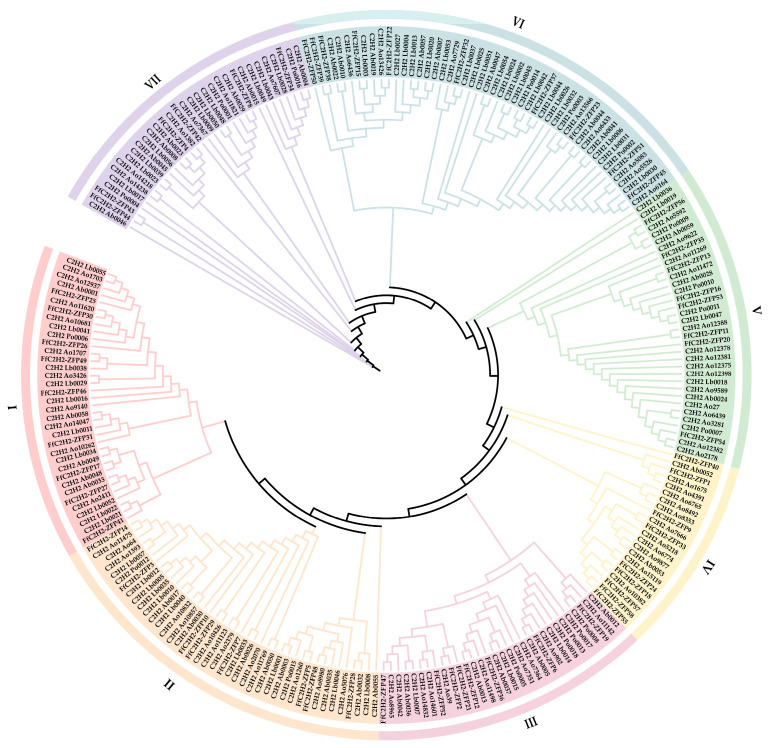
A phylogenetic tree of the C2H2-ZFPs among *F. filiformis*, *A. bisporus*, *L. bicolor*, *P. ostreatus*, and *A. ostoyae*. This tree was generated using the maximum likelihood method, and it displays seven distinct clades, each highlighted with different colors to denote their branches and ranges. The C2H2-ZFPs from *A. bisporus*, *L. bicolor*, *P. ostreatus*, and *A. ostoyae* are specifically labeled with the prefixes ‘C2H2 Ab’, ‘C2H2 Lb’, ‘C2H2 Po’, and ‘C2H2 Ao’, respectively.

To examine the evolutionary relationships of the C2H2-ZFPs in *F. filiformis* compared to those of the other fungi, we analyzed 58 C2H2-ZFPs from *F. filiformis*, 39 C2H2-ZFPs from *A. bisporus*, 57 C2H2-ZFPs from *L. bicolor*, 18 C2H2-ZFPs from *P. ostreatus*, and 72 C2H2-ZFPs from *A. ostoyae*. A phylogenetic tree was created employing the maximum likelihood approach, accompanied by a bootstrap support of 1000. Based on sequence similarity and phylogenetic tree topology, the C2H2-ZFPs were categorized into seven distinct groups: I, II, III, IV, V, VI, and VII. In *F. filiformis*, the distribution of the C2H2-ZFPs across these groups varied, with nine in group I, eight in group II, eight in group III, nine in group IV, eight in group V, ten in group VI, and eight in group VII (Figure 5). The percentages of the FfC2H2-ZFPs in *F. filiformis* within each group, relative to the total number of the C2H2-ZFPs in that group, were as follows: 25.71% for group I, 19.51% for group II, 28.81% for group III, 40.91% for group IV, 23.53% for group V, 20.41% for group VI, and 18.75% for group VII.

The analysis of the C2H2-ZFPs from five fungi indicates their presence in groups I, II, III, V, VI, and VII, suggesting that the differentiation of these fungi occurred after the initial diversification of the C2H2-ZFPs, except for group IV. Group IV primarily consists of proteins from *F. filiformis* (9) and *A. ostoyae* (12), with only two from *A. bisporus*, indicating the independent evolution of this group after main family diversification. Certain branches exhibit orthologous genes from different species, like FfC2H2-ZFP56 from *F. filiformis*, C2H2_Ao5592 from *A. ostoyae*, C2H2_Po0009 from *P. ostreatus*, and C2H2_Ab0059 from *A. bisporus* in group V, suggesting the conservation of similar functions across species. Some branches contain paralogous genes from the same species, for example, FfC2H2-ZFP38 and FfC2H2-ZFP39 in group VI and FfC2H2-ZFP43 and FfC2H2-ZFP44 in group VII, which are closely located on the chromosome, possibly evolving in response to different environmental pressures. The C2H2-ZFP gene family, with its significant gene count and complex evolutionary patterns, aligns with its diverse and crucial roles in biological systems.

### 3.6. Synteny Analysis of the FfC2H2-ZFP Genes

**Figure 6:**
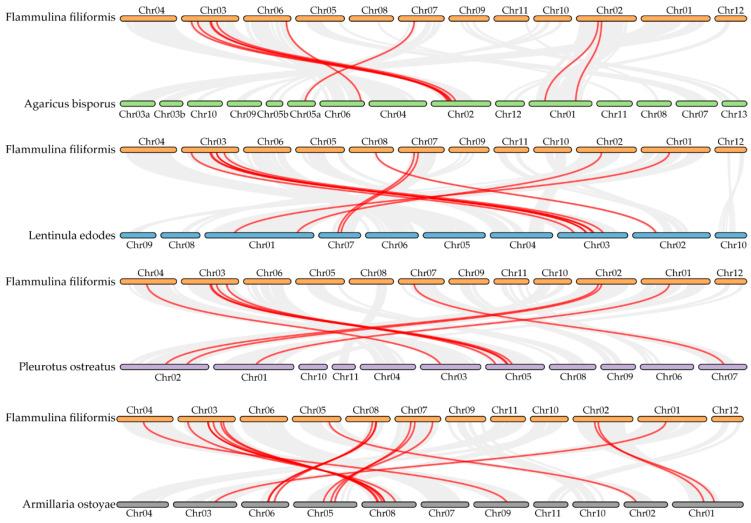
Synteny analyses of the *C2H2-ZFP* genes in *F. filiformis* compared to those of four other species. Collinear regions in *F. filiformis* are connected to those in other genomes using gray lines, with red lines indicating syntenic *C2H2-ZFP* gene pairs.

To investigate the evolutionary mechanisms of the FfC2H2-ZFPs, we studied four representative species from the order Agaricales, *A. bisporus*, *L. edodes*, *P. ostreatus*, and *A. ostoyae*, which belong to the families Agaricaceae, Omphalotaceae, Pleurotaceae, and Physalacriaceae, respectively. The *FfC2H2-ZFP* genes exhibited eight, nine, eight, and seventeen homologous gene pairs with these species, showing varying degrees of relatedness. Notably, *F. filiformis* and *A. ostoyae* displayed the closest relationship, which is consistent with their classification in the family Physalacriaceae. Moreover, specific *FfC2H2-ZFP* genes like *FfC2H2-ZFP6*, *FfC2H2-ZFP15*, and *FfC2H2-ZFP32* were conserved in *F. filiformis* and the other four edible fungi species. On the other hand, *FfC2H2-ZFP5*, *FfC2H2-ZFP12*, and *FfC2H2-ZFP14* were present in *F. filiformis* and three other species, suggesting their potential significance in the evolutionary history of the FfC2H2-ZFP gene family (Figure 6).

To gain further insights into the evolution of this gene family, we calculated Ka/Ks ratios for the homologous gene pairs. Most ratios could not be determined due to their high synonymous mutation probability (ps > 0.75), indicating significant sequence divergence. However, for the pairs where the Ka/Ks ratios were calculable, they were all less than one, indicating strong selective pressure and the likely removal of harmful mutations during the evolution of the *C2H2-ZFP* genes (Table S4).

### 3.7. Expression Pattern of the FfC2H2-ZFPs in Different Tissues

**Figure 7:**
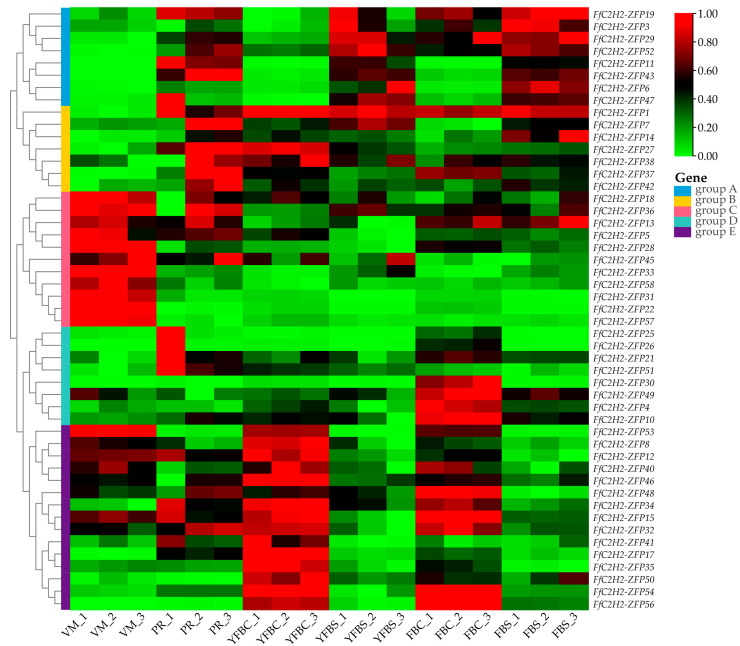
Expression profile analysis of the *FfC2H2-ZFP* genes across different tissues: vegetative mycelium (VM), primordium (PR), young fruiting body cap (YFBC), fruiting body cap (FBC), young fruiting body stipe (YFBS), and fruiting body stipe (FBS). FPKM values were normalized to a scale from zero to one.

To investigate the expression patterns of the FfC2H2-ZFP gene family, transcriptome data from 58 *FfC2H2-ZFP* genes in the various tissues of *F. filiformis* were analyzed. These tissues included the following: the vegetative mycelium (VM), the primordium (PR), the young fruiting body cap (YFBC), the fruiting body cap (FBC), the young fruiting body stipe (YFBS), and the fruiting body stipe (FBS). Analysis revealed distinct expression patterns among the different *FfC2H2-ZFP* genes, with most genes being expressed in all the tissues. Out of the 58 genes, 49 showed expression, with the FPKM values ranging from 1 to 619 (Figure 7, Table S5), while the remaining 9 genes had FPKM values below 1 in all the tissues, indicating non-expression. In light of the similarities observed in their expression profiles, the 49 expressed genes were divided into five distinct groups: Group A revealing higher expression levels in the primordia and stipe; Group B showing higher expression levels in the fruiting bodies; Group C demonstrating high expression levels at the mycelial stage; Group D displaying no significant changes in expression across the tissues; and Group E showing enhanced expression in the pileus.

**Figure 8:**
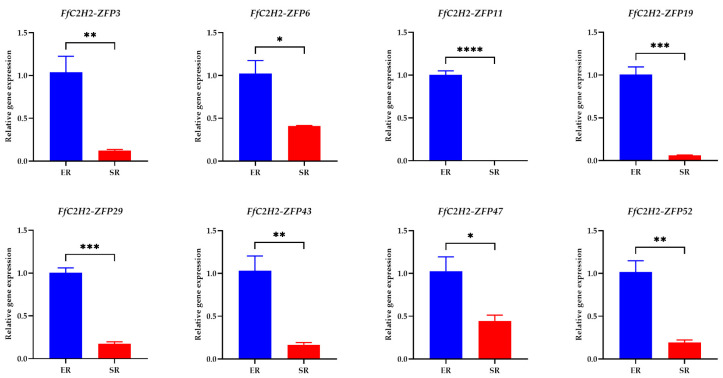
Relative expression levels of 8 *FfC2H2-ZFP* genes across samples with different stipe regions: elongation region (ER) and stable region (SR). Error bars represent the standard error of mean (SEM) calculated from three biological replicates. Asterisks indicate significant differences in gene expression following treatment (* *p* < 0.05, ** *p* < 0.01, *** *p* < 0.001, **** *p* < 0.0001; *t*-test).

**Figure 9:**
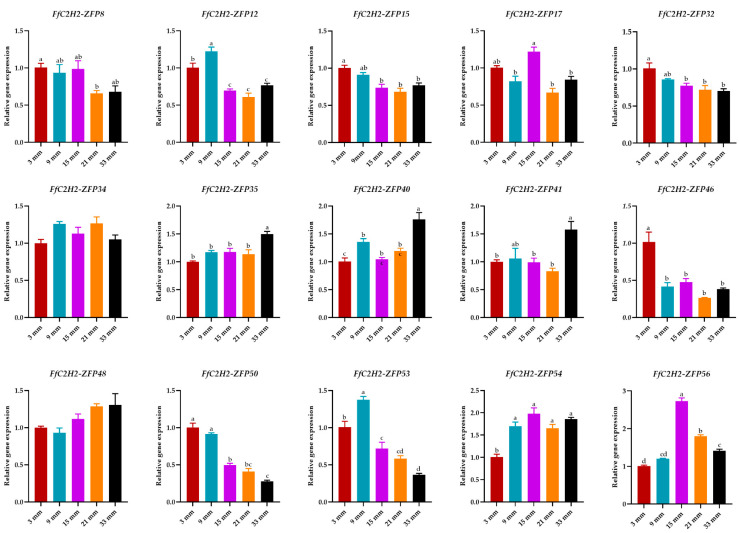
Relative expression levels of 15 *FfC2H2-ZFP* genes across samples with different pileus sizes: 3 mm, 9 mm, 15 mm, 21 mm, and 33 mm. Error bars represent the standard error of mean (SEM) calculated from three biological replicates. Different letters indicate significant differences in gene expression after treatment (*p* < 0.05; one-way ANOVA).

To further investigate the roles of the eight Group A genes in fruiting body development, qRT-PCR expression analysis was conducted on the different regions of the stipe. The elongation region (ER) consistently showed higher expression levels compared to those of the stable region (SR) (Figure 8). Furthermore, to validate the expression patterns of all of the 15 genes in Group E, qPCR analysis was performed across the pilei of varying sizes (Figure 9). The expression patterns exhibited notable diversity; for example, *FfC2H2-ZFP8* and *FfC2H2-ZFP12* displayed higher expression levels in the smaller pilei, while *FfC2H2-ZFP41* showed the highest expression level in the largest pilei. Interestingly, *FfC2H2-ZFP46* had the highest expression level in the smallest pileus, whereas *FfC2H2-ZFP54* had the lowest expression level at this stage. The expression levels of *FfC2H2-ZFP15*, *FfC2H2-ZFP32*, and *FfC2H2-ZFP50* tended to decrease as the pileus diameter expanded. Conversely, the levels of *FfC2H2-ZFP35*, *FfC2H2-ZFP40*, and *FfC2H2-ZFP48* generally increased. Notably, *FfC2H2-ZFP53* and *FfC2H2-ZFP56* initially showed an increase in expression, followed by a decline. The expression patterns of *FfC2H2-ZFP17* and *FfC2H2-ZFP34* did not exhibit a clear trend.

## 4. Discussion

The C2H2-ZFPs are one of the largest families of transcription factors found in eukaryotic genomes [44]. Our research presents the first comprehensive analysis of C2H2 zinc finger proteins (C2H2-ZFPs) in *F. filiformis*, identifying 58 distinct FfC2H2-ZFP members characterized by unique sequence features and specific chromosomal locations. This discovery enhances the existing knowledge of the C2H2-ZFPs in edible fungi, which have previously been associated with the development of other species, such as *P. ostreatus* and *A. bisporus*. The expression patterns of these genes during various developmental stages, as revealed by our RNA-seq and qRT-PCR analyses, indicate their critical roles in the morphogenesis of *F. filiformis*. These findings not only address the gap in the literature but also provide new opportunities for investigating the molecular mechanisms that underlie the development of fruiting bodies in *F. filiformis*.

Understanding sequence characteristics, gene structures, and subcellular localization is essential for determining gene function [45,46]. Despite the variations in molecular weight, all the proteins of the C2H2-ZFP gene family in *F. filiformis* were found to be localized in the nucleus, which is consistent with their role as transcription factors. The C2H2-ZFP gene family in *F. filiformis* was classified into nine subfamilies based on the differences in protein sequences, with each subfamily showing similar protein sequences and closely related exon–intron gene structures. The number and distribution of introns, which have been conserved through evolutionary processes, play a role in gene expression and transcriptional regulation. Further detailed investigations are required to elucidate the specific mechanisms of their function.

Gene duplication significantly contributes to the redundancy and divergence of gene functions during genomic evolution [47]. In *F. filiformis*, the absence of duplicated *C2H2-ZFP* genes and the relatively low number of homologous gene pairs with other fungi, such as the 17 pairs (29.3%) identified in *A. ostoyae* from the same family, suggest that *F. filiformis* has undergone fewer gene duplication events in its evolutionary history, highlighting the substantial variations among fungi. Conversely, plants exhibit more collinear gene pairs. For instance, in *Sorghum bicolor*, 34 out of the 145 *C2H2-ZFP* genes are recognized as duplicates. Some noteworthy examples include 203 homoeologous gene pairs with *Zea mays* and 123 with *Oryza sativa*, which are both from the same family, and 85 with *Glycine max* as well as 23 with *Arabidopsis thaliana*, which belong to different orders from Sorghum [11]. Similar trends have been observed in the Q-type C2H2 gene family of *Solanum tuberosum* [48], the *C2H2-ZFPs* of *Opisthopappus* [49], and the *FTIP* family of *O. sativa* [50]. In comparison to plants, fungi demonstrate a lower frequency of gene duplication and homologous genes throughout their evolutionary history, while exhibiting significantly greater diversity. This disparity may be linked to their responses to varying environmental pressures. Further research is necessary to elucidate the specific factors that drive these evolutionary patterns.

The distribution and variety of cis-regulatory elements in promoter sequences play a critical role in gene expression and functionality [51]. The *FfC2H2-ZFP* genes are rich in elements associated with development, hormonal regulation, and stress responses, such as the G-, CAT-, and A-boxes; the CGTCA, TGACG, ARE, MBS, and LTR motifs; and the ABRE and TGA elements. These elements make up 94.95% of the total cis-element count, underscoring the significance of the FfC2H2-ZFPs in the growth, development, and stress resilience of *F. filiformis*. This discovery aligns with the known functions of the C2H2-ZFPs in other species.

Gene expression levels are closely linked to biological functions. The previous studies have shown that the *C2H2-ZFP* genes exhibit significant variations in expression among different tissues of various species. Our findings reveal distinct expression differences in various tissues for the *FfC2H2-ZFP* genes, which are associated with the growth characteristics of *F. filiformis*. Particularly noticeable disparities are observed during the mycelial and fruiting body stages as well as between the stipe and pileus of the fruiting body. The differential expression of the *FfC2H2-ZFP* genes indicates their diverse roles in *F. filiformis* fruiting body development. The examination of the stipe revealed distinct expression levels, while the analysis of pilei of varying sizes revealed multiple gene expression patterns. Previous research often oversimplified developmental stages, such as the mycelium, the primordium, the stipe, the pileus, and the elongation and maturation stages, overlooking more detailed spatial and temporal analyses that could potentially mask the true functions of genes. For instance, *FfC2H2-ZFP56* shows an initial increase, followed by a decrease in expression as the pileus grows. However, this pattern may not be apparent in samples with limited tissue, where subtle gene expression trends could be obscured. Therefore, conducting more thorough research on tissue expression over time and across different regions can improve our understanding of gene functions.

## Data Availability

The original contributions presented in the study are included in the article/Supplementary Material, further inquiries can be directed to the corresponding authors.

## Supplementary Materials

The following supporting information can be downloaded at https://www.mdpi.com/article/10.3390/jof10090644/s1, Figure S1: The synteny analysis of the *FfC2H2-ZFP* genes. The gray lines indicate collinear regions in the *F. filiformis* genome. The outer circle shows the chromosome numbers, and the subsequent circles represent gene density across the chromosomes; Figure S2: Motif sequences identified in the FfC2H2-ZFPs of *F. filiformis*; Figure S3: Alignment of C2H2 domains across the FfC2H2-ZFPs; Figure S4: The identification of the predicted cis-acting elements within the promoter regions extending 2000 base pairs upstream of the *FfC2H2-ZFP* genes; Table S1: Gene-specific primer pairs employed for qRT-PCR; Table S2: Information on the C2H2-ZFPs in *F. filiformis*; Table S3: The sequences of the 58 FfC2H2-ZFP genes identified in this study; Table S4: Ka/Ks values for collinear gene pairs between *F. filiformis* and four other representative species; and Table S5: FPKM values of 58 *FfC2H2-ZFP* genes.
