# Supplementary figures and images for: Genome-Wide Identification and Expression Analysis of the Cys2His2 Zinc Finger Protein Gene Family in Flammulina filiformis
Source: J Fungi (Basel). 2024 Sep 11;10(9):644. doi: 10.3390/jof10090644 (PMC11433517; doi:10.3390/jof10090644)

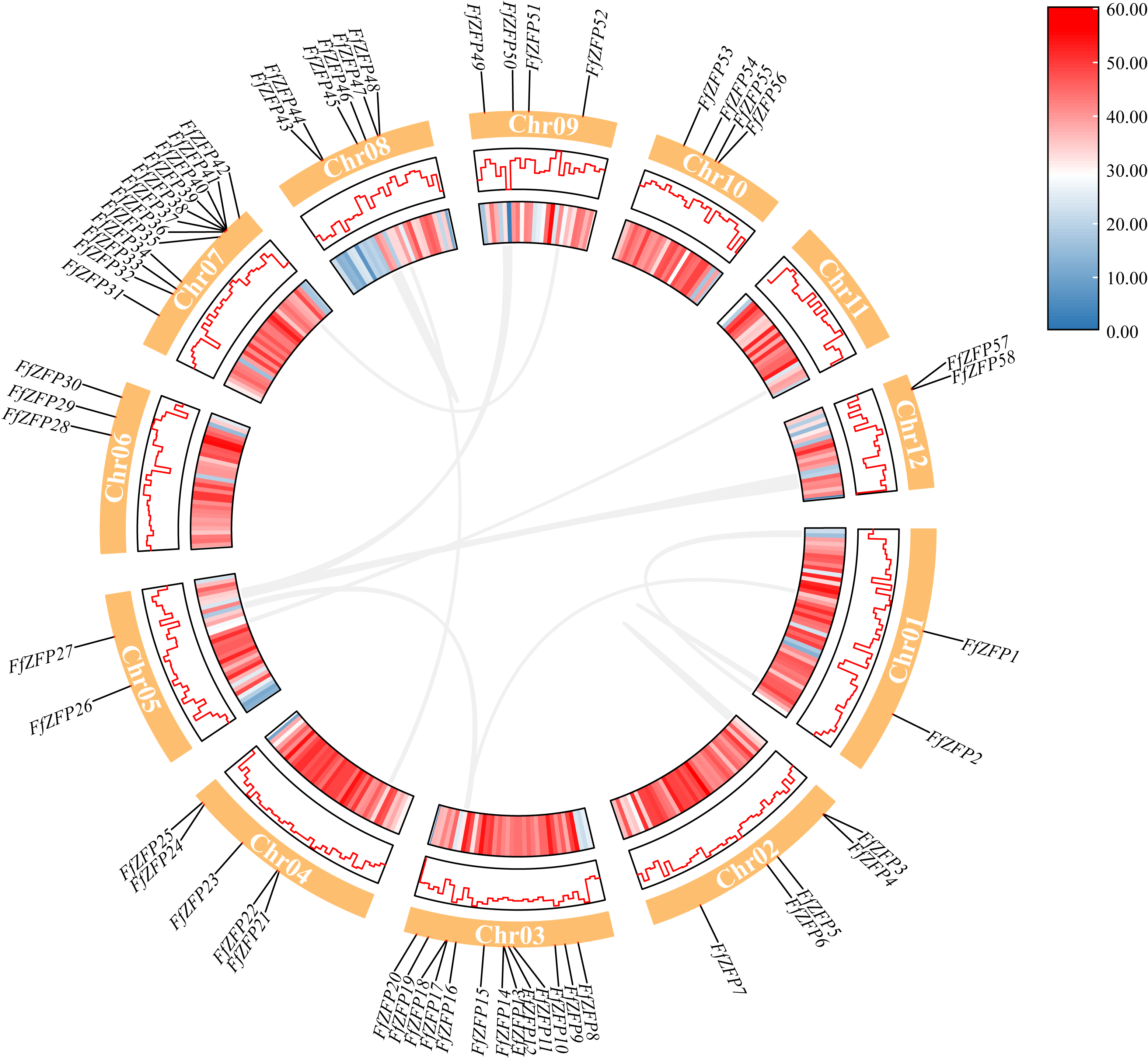

Supplement: Supplementary file 1 [file jof-10-00644-s001.zip › Figure S1 Synteny analysis of FfC2H2 ZFP genes.tif]

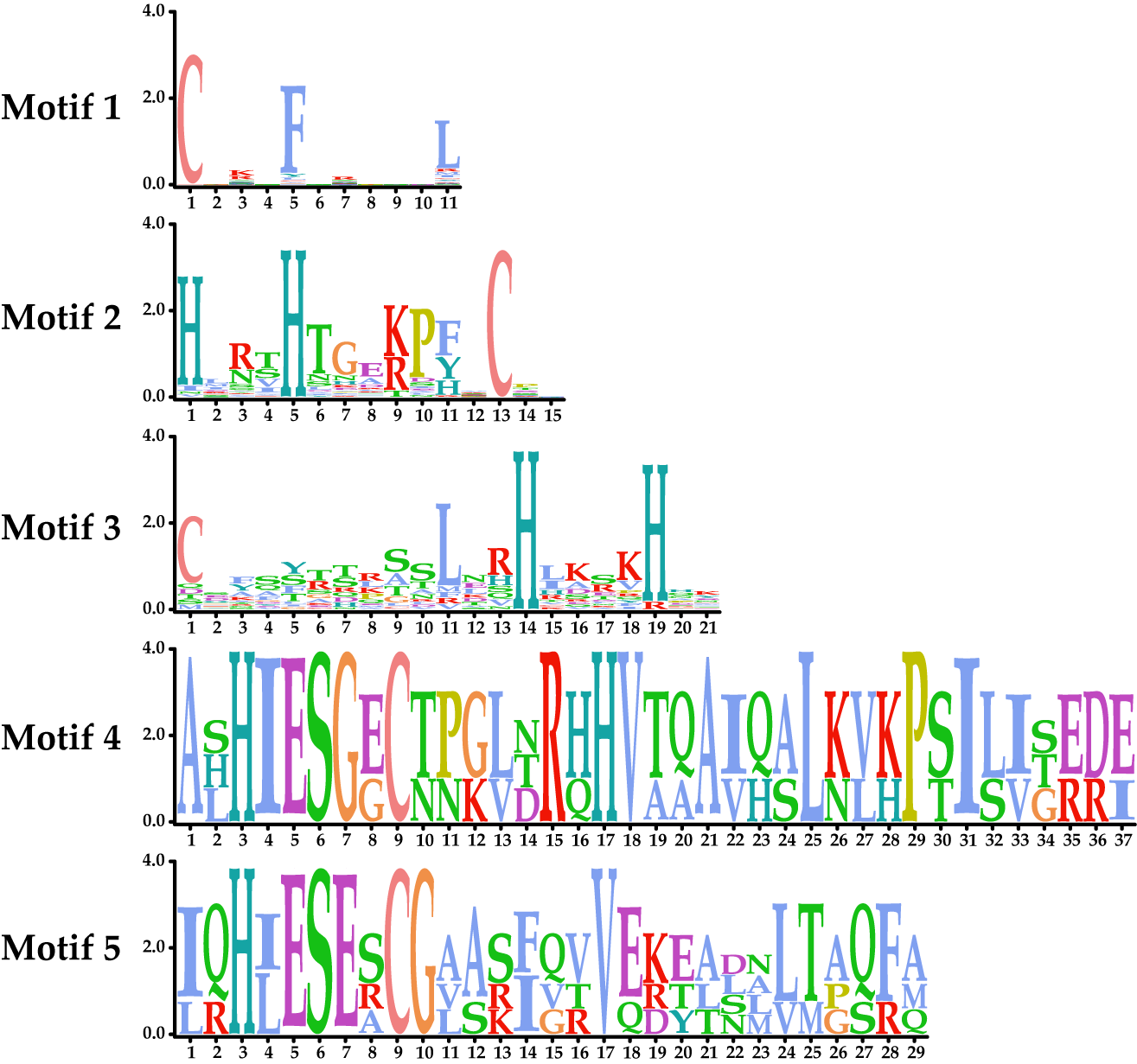

Supplement: Supplementary file 1 [file jof-10-00644-s001.zip › Figure S2 Motif sequences identified in FfC2H2-ZFPs of F. filiformis.tif]

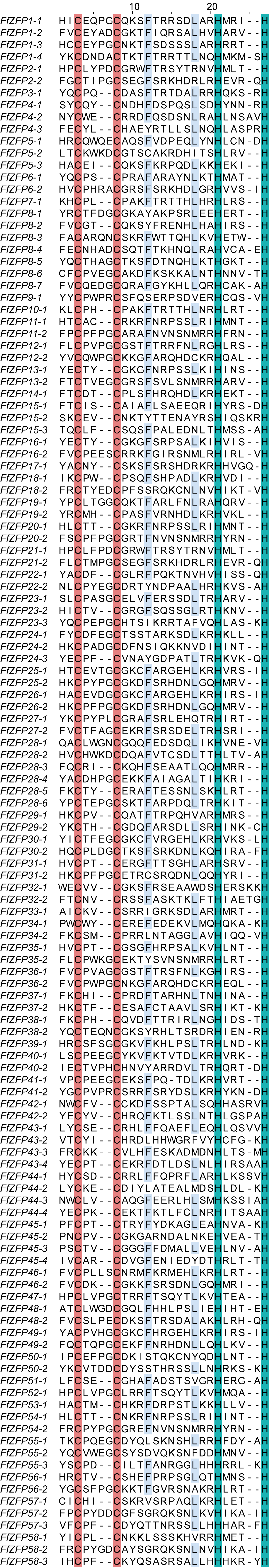

Supplement: Supplementary file 1 [file jof-10-00644-s001.zip › Figure S3 Alignment of C2H2 domains across FfC2H2-ZFPs.tif]

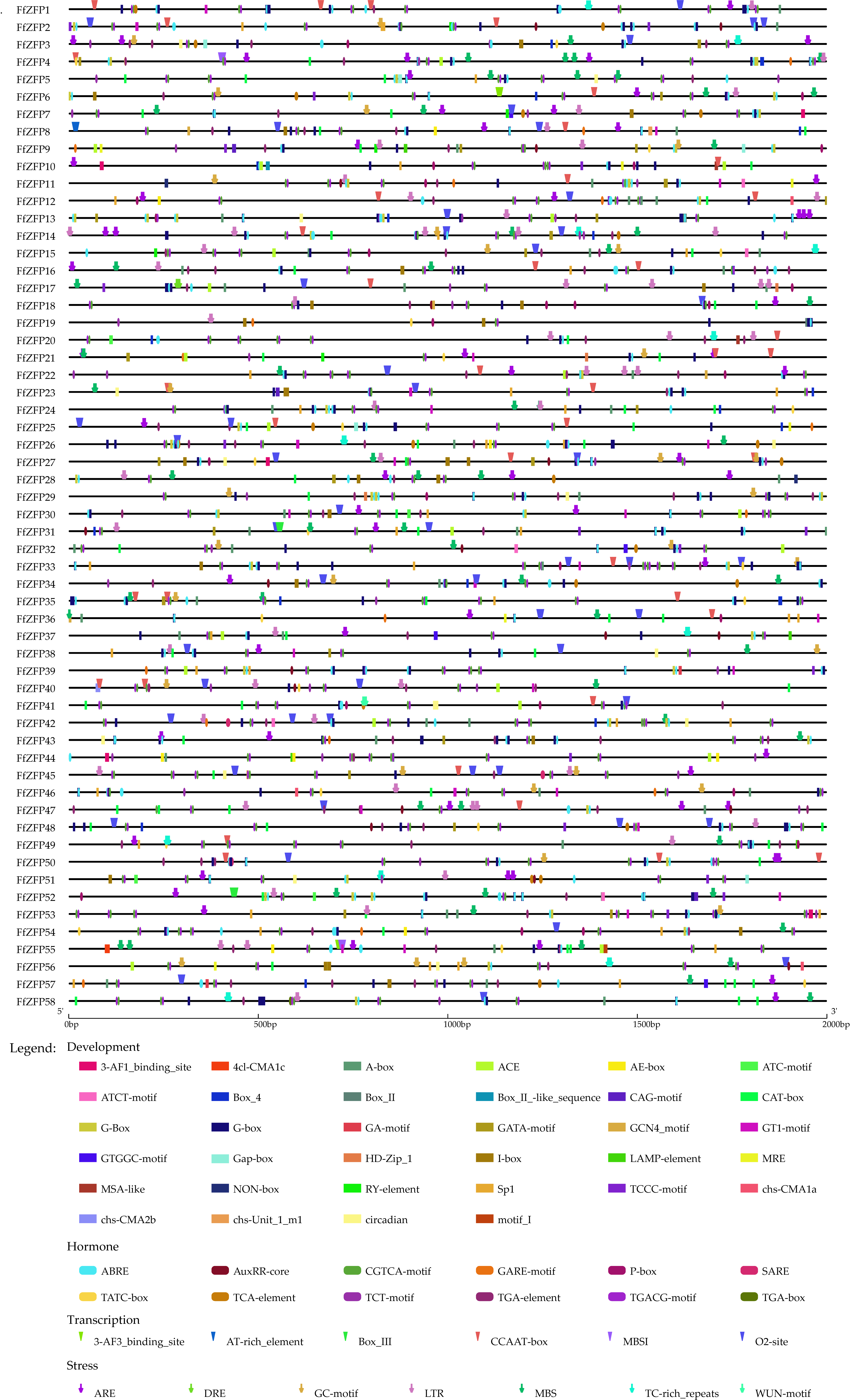

Supplement: Supplementary file 1 [file jof-10-00644-s001.zip › Figure S4 The identification of the predicted cis-acting elements within the promoter regions extending 2000 base pairs upstream of the FfC2H2-ZFP genes.tif]
